# Characterization of the DdrD protein from the extremely radioresistant bacterium *Deinococcus radiodurans*

**DOI:** 10.1007/s00792-021-01233-0

**Published:** 2021-05-29

**Authors:** Claire Bouthier de la Tour, Martine Mathieu, Pascale Servant, Geneviève Coste, Cédric Norais, Fabrice Confalonieri

**Affiliations:** 1 Institute for Integrative Biology of the Cell (I2BC), CEA, CNRS, Université Paris-Saclay, 91198 Gif sur Yvette, France; 2 Department of Biochemistry, University of Wisconsin, Madison, WI 53706 USA; 3 Present Address: SAT Lyon, Promega France, 24 Chemin des Verrieres, 69260 Charbonnières les Bains, France

**Keywords:** *Deinococcus radiodurans*, DdrD, DNA binding protein, DNA damage response

## Abstract

Here, we report the in vitro and in vivo characterization of the DdrD protein from the extraordinary stress-resistant bacterium, *D. radiodurans*. DdrD is one of the most highly induced proteins following cellular irradiation or desiccation. We confirm that DdrD belongs to the Radiation Desiccation Response (RDR) regulon protein family whose expression is regulated by the IrrE/DdrO proteins after DNA damage. We show that DdrD is a DNA binding protein that binds to single-stranded DNA In vitro, but not to duplex DNA unless it has a 5′ single-stranded extension. In vivo, we observed no significant effect of the absence of DdrD on the survival of *D. radiodurans* cells after exposure to γ-rays or UV irradiation in different genetic contexts. However, genome reassembly is affected in a *∆ddrD* mutant when cells recover from irradiation in the absence of nutrients. Thus, DdrD likely contributes to genome reconstitution after irradiation, but only under starvation conditions. Lastly, we show that the absence of the DdrD protein partially restores the frequency of plasmid transformation of a *∆ddrB* mutant, suggesting that DdrD could also be involved in biological processes other than the response to DNA damage.

**Supplementary Information:**

The online version contains supplementary material available at 10.1007/s00792-021-01233-0.

## Introduction

*Deinococcus radiodurans* is well known for its extreme resistance to radiation, desiccation and various DNA-damaging chemicals such as mitomycin C and hydrogen peroxide. Data from various studies (Ishino and Narumi 2015; Slade and Radman 2011) strongly suggested that the radioresistance of *D. radiodurans* is a combination of multiple strategies, including protection of proteins against oxidation, efficient DNA repair pathways, a condensed nucleoid structure favoring the maintenance of DNA fragment cohesion after irradiation.

Global analysis of *D. radiodurans* genome expression allowed the identification of a series of genes whose expression is induced after irradiation or desiccation (Tanaka et al. 2004). Most of the highly induced genes encode proteins involved in DNA repair (RecA, RuvB, UvrA, UvrB, UvrD), DNA supercoiling (GyrA and GyrB) as well as several deinococcal specific proteins (PprA, DdrA, DdrB, DdrC, DdrD) involved in the response to DNA damage. All these genes contain a 17 bp RDRM (Radiation Desiccation Response Motif) sequence in their promoter region (Makarova et al. 2007), a hallmark of a set of genes identified as members of a radiation/desiccation response (RDR) regulon. It was previously shown that the expression of predicted RDR proteins in *D. radiodurans* like PprA*,* GyrA*,* DdrB, and DdrC, is regulated by the couple of IrrE and DdrO proteins after DNA damage (de la Tour et al. 2017; Devigne et al. 2015). DdrO binds to the RDRM sequence and acts as a repressor of the RDR regulon (Blanchard et al. 2017; Wang et al. 2015). After irradiation, IrrE stimulated by an increased availability of zinc ions (Blanchard et al. 2017), is able to cleave the DdrO repressor, then leading to the derepression of the RDR genes.

The PprA, DdrA, DdrB, DdrC, and DdrD proteins are recruited to the nucleoid early after exposure to γ-irradiation (de la Tour et al. 2011, 2013,2017; Devigne et al. 2013). The PprA, DdrA, DdrB, and DdrC proteins have been well characterized and were proposed to be part of the *D. radiodurans* genome-protection system. They are DNA binding proteins that exhibit various and redundant activities. PprA protein preferentially binds to double-stranded DNA (dsDNA) ends, stimulates in vitro DNA ligase activity (Narumi et al. 2004) and in vivo was found to be involved in chromosome segregation (Devigne et al. 2013, 2016; Kota et al. 2014a, b). DdrB is an SSB-like protein (Norais et al. 2009) that binds to single-stranded DNA (ssDNA) and stimulates annealing of complementary ssDNA. DdrB participates in the early stages of DNA double strand break repair through a single strand annealing (SSA) mechanism when cells are exposed to high levels of irradiation (de la Tour et al. 2011; Xu et al. 2010). DdrA preferentially binds to 3′ ssDNA ends and protects them from nuclease degradation, suggesting that it contributes to the preservation of genome integrity after irradiation (Harris et al. 2004). More recently, we have shown that DdrC is a DNA binding protein that binds single and double-stranded DNA with a preference for the ssDNA, protects DNA from nuclease attack and exhibits a DNA strand annealing activity (de la Tour et al. 2017). It was suggested that DdrC maintains DNA fragments end to end, thus limiting dispersion and extensive degradation after exposure to high doses of radiation. However, while the properties of PprA, DdrA, DdrB, and DdrC proteins are well documented, little is known about the DdrD protein.

Here, we investigated the in vitro and in vivo properties of the DdrD protein to gain a better understanding of its potential role in irradiated cells. We showed that the expression of DdrD is induced after γ-irradiation and is under the control of the IrrE/DdrO system. In vitro, the DdrD protein binds to ssDNA and to dsDNA with a single-stranded 5′ extension. Although it does not protect DNA from nuclease attack, we showed that its absence alters genome reconstitution after *D. radiodurans* cells were irradiated and recovered in a nutrient-poor environment. We also re-examined the effects in vivo of associated deletions of the *ddrA, ddrB*, *ddrC*, and *ddrD* genes on the cellular response to exposure to γ-rays and to UV irradiation. For this purpose, we constructed all possible double, triple, and quadruple mutants. Analysis of the resulting strains revealed no significant effect of the *ddrD* deletion, even if this deletion was associated with deletions of *ddrA, ddrB, and ddrC* genes. Finally, we showed that the absence of DdrD partially suppresses the impact of the deletion of the *ddrB* gene on plasmid transformation suggesting that the DdrD protein, like other Ddr proteins, may be involved in several different biological processes.

## Materials and methods

### Bacterial strains, plasmids, and growth conditions

Bacterial strains and plasmids used in this study are listed in Table 1. To construct *D. radiodurans* deletion mutants or strains expressing a recombinant tagged protein, the loci of interest were replaced with the appropriate antibiotic resistance cassette or their tagged counterparts, respectively, using the tripartite ligation method (Mennecier et al. 2004). The double mutants were constructed by transformation of a single mutant by the genomic DNA of another single mutant. The same strategy was used for the construction of triple and quadruple mutants. Genomic DNA of *D. radiodurans* was purified and transformation of *D. radiodurans* with PCR products or genomic DNA was performed as previously described (de la Tour et al. 2011). The genetic structure and the purity of mutant strains were verified by PCR. Oligonucleotides used for constructions of mutants, diagnostic PCR, and sequencing are available upon request.

**Table 1:** Bacterial strains and plasmids

Bacterial strains *D. radiodurans*	Description	Source or references
R1/GY9613	Wild type, ATCC 13939	Laboratory stock
GY 15921	*ddrD::HA-kan*	de la Tour et al. (2013)
GY 15951	*ddrD::HA-kan ∆irrEΩcat*	This work
GY14164	*ΔddrOΩcat/p11891(prepUTs::ddrO*^+^*)*	Devigne et al. (2015)
GY16922	[*ddrD::HA-kan ΔddrOΩcat/p11891(prepUTs::ddrO*^+^*)*]	This work
GY15924	*∆ddrDΩkan*	This work
GY15929	*∆ddrCΩkan*	de la Tour et al. (2017)
GY15930	*∆ddrCΩcat*	This work
GY 16936	*∆ddrCΩhygro*	This work
GY 16002	*∆ddrAΩcat*	Laboratory stock
GY16926	*∆ddrAΩhygro*	This work
GY13915	*∆ddrBΩcat*	Laboratory stock
GY 12835	*∆ddrBΩkan*	de la Tour et al. (2011)
GY16938	*∆ddrBΩtet*	This work
GY16944	*∆ddrAΩhygro∆ddrBΩkan*	This work
GY16931	*∆ddrAΩhygro∆ddrCΩkan*	This work
GY16932	*∆ddrAΩhygro ∆ddrDΩkan*	This work
GY15937	*∆ddrBΩcat ∆ddrCΩkan*	This work
GY15936	*∆ddrBΩcat ∆ddrDΩkan*	This work
GY15932	*∆ddrCΩcat ∆ddrDΩkan*	This work
GY16933	*∆ddrAΩhygro∆ddrCΩcat ∆ddrDΩkan*	This work
GY16934	*∆ddrAΩhygro ∆ddrBΩcat ∆ddrCΩkan*	This work
GY16935	*∆ddrAΩhygro ∆ddrBΩcat ∆ddrDΩkan*	This work
GY16937	*∆ddrBΩcat ∆ddrCΩhygro ∆ddrDΩkan*	This work
GY16938	*∆ddrAΩhygro ∆ddrBΩtet ∆ddrCΩcat ∆ddrDΩkan*	This work
GY15971	*∆uvrAΩcat*	de la Tour et al. (2017)
GY15972	*∆uvsEΩhygro*	de la Tour et al. (2017)
GY15975	*∆ddrDΩkan ∆uvrAΩcat*	This work
GY15976	*∆ddrDΩkan ∆uvsEΩhygro*	This work
GY12251	*∆pprAΩcat*	Devigne et al. (2013)
GY15934	*∆pprAΩcat ∆ddrDΩkan*	This work

Plasmids	Description	Source or references
p11086	Source of *kan* cassette	Laboratory stock
pPS6	Source of *cat* cassette	Laboratory stock
p12625	Source of *hygro* cassette	Laboratory stock
P11165	Source of *tet* cassette	Laboratory stock
p12764	Source of *HA::kan* cassette	Laboratory stock
p11891	p13841*:*prep*UTs::ddrO*^+^, *spec*^*R*^	Devigne et al. (2015)
p11559	Shuttle vector *E. coli*/*D. radiodurans*, Spec^R^	Laboratory stock
pET21	Expression vector	Novagen
pEAW21	pET21–*DdrD*	This work

*D. radiodurans* bacteria were grown at 30 °C in TGY2X (1% tryptone, 0.2% dextrose, 0.6% yeast extract) or plated on TGY1X containing 1.5% agar. Media were supplemented with the appropriate antibiotics used at the following concentrations: hygromycin, 50 μg/mL; chloramphenicol, 3.5 μg/mL; kanamycin, 6 μg/mL; tetracycline, 2.5 µg/mL, and spectinomycin, 75 µg/mL. *E. coli* was grown in Luria–Bertani medium at 37 °C with the appropriate antibiotics to the following concentrations: ampicillin, 100 µg/mL; kanamycin, 40 µg/mL.

Transformation of *D. radiodurans* with plasmid DNA was performed as previously described (de la Tour et al. 2011).

### Expression and purification of DdrD protein

The gene coding for the DR0326 protein, as indicated in Genbank, was amplified from *D. radiodurans* genomic DNA by PCR using primers DR0326us (GGAACAGCATATGGATACCCTGAAAAAAGCTGGAACGATGC) and DR0326ds (GGAATTCTTAGGCTGCCGGGGTGTTTTCGCCGGCCTCGCTC). The resulting product was inserted into the *Nde*I and the *Eco*RI cloning sites of pET21a (Novagen) to yield construct pEAW321. The construct was transformed into the *E. coli* expression strains STL2669 pT7pol26 [*Δ(recA-srlR)306::Tn10 xonA2(sbcB*^*−*^), a gift from Susan T. Lovett (Brandeis University, Waltham, MA). pT7pol26 is described in (Lusetti et al. 2003). The cells were grown in 10 L LB broth containing 100 µg/mL ampicillin and 40 µg/mL kanamycin at 37 °C to an OD_600 nm_ of 0.5. Overexpression of DdrD was then induced with 0.4 mM IPTG (GoldBio) and grown at 37 °C for three more hours before harvest. The 13 g cell pellet was frozen in liquid nitrogen and thawed overnight at 4 °C in 50 mL of R buffer (20 mM Tris–Cl 80% cations, 100 µM EDTA, and 10% w/v glycerol). All subsequent steps were performed at 4 °C. Lyzozyme (Sigma) was added to a final concentration of 0.2 mg/mL. Cells were stirred for 2 h and then sonicated on ice. Insoluble material and cell debris were pelleted and removed by centrifugation at 38,000*g* for 2 h and the cell lysate supernatant was precipitated by the dropwise addition of 10 mL of 5% w/v polyethyleneimine. The solution was stirred for 1 h, then centrifuged for 15 min at 9000*g*. The protein remained in the supernatant. The supernatant was brought to 30% NH_4_(SO_4_)_2_ (MP Biochemical) by addition of solid powder (15 g NH_4_(SO_4_)_2_ to 85 mL). The solution was stirred 1 h, then centrifuged for 30 min at 25,000*g*. The pellet was discarded and the remaining supernatant brought to 40% saturation by additional NH_4_(SO_4_)_2_ (5.49 g NH_4_(SO_4_)_2_ to 90 mL). The solution was stirred for 4 h and centrifuged for 30 min at 25,000*g*. The DdrD protein remained in the pellet and was eluted from the pellet using R buffer containing 1 M NH_4_(SO_4_)_2_. The resuspended protein was then loaded on a 120 mL Butyl Sepharose (Amersham) column using an AKTA FPLC system. DdrD bound to the butyl column and was eluted by a gradient from R buffer containing 1 M NH_4_(SO_4_)_2_ to R buffer only, through 10 column volumes. The protein eluted at a concentration of around 700 mM NH_4_(SO_4_)_2_ in buffer R. Fractions containing DdrD were pooled (90 mL) and brought to 50% NH_4_(SO_4_)_2_ saturation by the addition of 38 g of NH_4_(SO_4_)_2_. The solution was centrifuged at 25,000*g* for 30 min, and the pellet resuspended in 10 mL R buffer. The resuspended protein solution was dialyzed 4 times 2 h against R buffer and loaded on a 25 mL DEAE column. DdrD binds poorly to the DEAE column and was recovered in the flow through in a cleaner state. The flow through containing DdrD was dialyzed in R buffer and loaded on a 20 mL SP Sepharose column. DdrD binds poorly to the SP Sepharose and was recovered from the flow through. The 75 mL flow through was dialyzed 4 times against 2 L of P buffer (20 mM phosphate buffer) and loaded onto a 20 mL hydroxyapatite (HAP) column. A gradient of five column volumes to reach 1 M phosphate buffer was applied. DdrD also binds poorly to the HAP, the flow through was dialyzed 4 times 2 h against 2 L of R buffer + 1 M NH_4_(SO_4_)_2_. DdrD was then loaded onto a 120 mL Butyl column, one column volume was applied to wash the column to elute and concentrate the protein in one step by going directly to R buffer. The elution fractions (~ 120 mL) were pooled and concentrated to 5 mL with a Centricon Plus 20 (Merck Millipore). The protein concentration was determined by measuring the absorbance at 280 nm and using the calculated extinction coefficient *ε*_DdrD_ = 5120 M^−1^ cm^−1^ (0.2420 (mg/mL)^−1^ cm^−1^). It was estimated at 5.4 mg/mL or 254 µM. Mass spectrometry analysis (MALDI–TOF) confirmed the purified protein was the expected 21.1 kDa *D. radiodurans* DR0326 DdrD. The purified protein was free of any detectable nuclease activity. The undiluted protein was flash frozen in 20 µL aliquots and stored at −80 °C.

### Glutaraldehyde treatment

Purified DdrD and DdrC proteins were diluted in a buffer containing 50 mM Tris–HCl pH 8, 15% (V/V) glycerol, and 1 mM DTT. They were incubated with 0.1% glutaraldehyde in 10 mM sodium phosphate buffer (pH 7) at 30 °C for 30 min in a final volume of 20 µL. After incubation, 5 µL of 5X Laemmli buffer (312.5 mM Tris–HCl pH 6.8, 50% glycerol, 10% SDS, 250 mM DTT, 0.1% bromophenol blue) were added and the samples were analyzed by electrophoresis through a 15% SDS–polyacrylamide gel, followed by Coomassie blue staining. DdrC protein used as a positive control is a gift of J. Timmins (Univ Grenoble Alpes, France),

### γ-irradiation of *D. radiodurans* bacteria

A saturated bacterial culture was diluted in fresh TGY2X medium and incubated at 30 °C to an *A*_650 nm_ = 0.3. Cells were then concentrated to *A*_650 nm_ = 20 by centrifugation and exposed to 5 kGy or 8 kGy γ-irradiation on ice (^137^Cs irradiation system GSR-D1, dose rate 18.5 Gy/min, Institut Curie, Orsay). Following irradiation, diluted cells were plated on TGY plates. Colonies were counted after 3–5 days incubation at 30 °C.

### UV irradiation of *D. radiodurans* bacteria

The UV sensitivity of *D. radiodurans* bacteria was tested on plates. Cultures of exponentially growing cells at an *A*_650 nm_ = 0.3 were serially diluted 1:10 in TGY2X broth and aliquots (10 µL) of each dilution were spotted on TGY1X agar plates. The plates were exposed to different doses of UV radiation using a UV-C lamp emitting at a calibrated dose rate of 3.5 J m^−2^ s^−1^ and incubated at 30 °C for 3–5 days.

### Western blot analysis of HA-tagged DdrD protein

Non-irradiated or irradiated cultures (5 kGy) of *D radiodurans* producing the DdrD-HA protein were diluted in 120 mL TGY2X broth to an *A*_650 nm_ = 0.2 and incubated at 30 °C with shaking. Aliquots of 15 mL were taken at different times and centrifuged. The cell pellets were resuspended in 150 µL 1X SSC buffer (150 mM NaCl, 15 mM trisodium citrate, pH 7) and cell extracts were prepared as previously described (de la Tour et al. 2009). 10 µg of crude extracts were resolved in 12% SDS–PAGE gels and transferred onto a PVDF membrane (GE Healthcare). The membranes were incubated overnight at 4 °C with a 1:5000 dilution of monoclonal mouse anti-HA antibodies (Eurogentec), and then 1 h at room temperature with a secondary alkaline phosphatase-labeled anti-mouse antibody, and revealed by a colorimetric reaction using nitroblue tetrazolium (NBT) and 5-bromo-4-chloro-3-indolyl phosphate (BCIP) as substrates for the alkaline phosphatase (Promega).

### DdrO depletion

*D. radiodurans* strain GY16922 [*ddrD::HA-kan* Δ*ddrOΩcat*/*p11891*(*prepU*_*Ts*_* ddrO*^+^)] was grown at a permissive temperature (30 °C) in TGY2X medium supplemented with chloramphenicol and spectinomycin. Cultures at an *A*_650_ = 0.3 were centrifuged and cell pellets were resuspended in the same volume of fresh culture medium supplemented with chloramphenicol. Then, cells were grown at permissive (30 °C) or non-permissive (37 °C) temperature to allow replication or not of the repU_Ts_ plasmid. Aliquots of 20 mL were taken for western blot analyses after 4 h, 8 h, and 16 h incubation.

### Electrophoretic mobility shift assay (EMSA)

Synthesized DNA substrates were purchased from Integrated DNA Technologies or Eurofins. The sequence of the 67-mer oligonucleotide, arbitrarily selected from the M13 phage genome, was 5′-CTGTTTAAGAAATTCACCTCGAAAGCAAGCTGATAAACCGATACAATTAAAGGCTCCTTTTGGAGCC-3′. The sequence of the corresponding 67 mer reverse oligonucleotide was: 5′-GGCTCCAAAAGGAGCCTTTAATTGTATCGGTTTATCAGCTTGCTTTCGAGGTGAATTTCTTAAACAG-3′.

Binding of DdrD protein to oligonucleotides was performed using a single-stranded 5′ Cy5-labeled 67 mer oligonucleotide (oligo 1) or the corresponding double-stranded 67 mer substrate (oligo 2). To generate the ds 67 mer substrate (oligo 2), 1 pmol of labeled 67 mer oligonucleotide and 1 pmol of 67 mer reverse oligonucleotide were mixed together in a buffer (20 mM Tris–HCl pH 7.5, 50 mM NaCl), heated at 95 °C for 2 min, and cooled for 2 h at room temperature. Double-stranded substrates with a 37-nt 5′ tail (oligo 3) or with a 37-nt 3′ tail (oligo 4) were generated by annealing oligo 1 with either the 30-mer oligonucleotide (5′-GGCTCCAAAAGGAGCCTTTAATTGTATCGG-3′) or the 30-mer oligonucleotide (5′-GCTTGCTTTCGAGGTGAATTTCTTAAACAG-3′), respectively. All reactions were performed in 15 µL of buffer A (40 mM Tris–HCl pH 7.8, 5 mM MgCl_2_, 1.5 mM DTT, 50 mM NaCl, 2.5% glycerol) containing 50 fmoles (3.3 nM) of DNA. The reaction was initiated by adding the DdrD protein at the indicated final concentrations. The mixture reaction was incubated at 4 °C for 15 min and loaded onto 6% (w/v) native polyacrylamide gels (19:1 (w/w) acrylamide/bisacrylamide) in 0.25X TBE buffer (Tris/Borate/EDTA) containing 10% (V/V) glycerol. The gels were pre-run before loading the reaction mixtures. After migration at 15 V/cm for 135 min at 4 °C, bands were visualized by scanning with a Typhoon phosphorimager (Typhoon Trio Imager, GE Healthcare).

### Nuclease protection assays

The assays were performed with 1 U DNase I (Promega) or 30 U RecJ (New England Biolabs). 3.3 nM of double-stranded oligonucleotide with the 37-nt 5′ tail (oligo 3) was used in the nuclease assays with DNase I. For the assay with RecJ, the 3′ Cy5-labeled 67-mer oligonucleotide was used to generate the corresponding dsDNA substrate (oligo 5). DNA was pre-incubated with DdrD protein (8 µM) for 15 min at 4 °C in 16 µL of buffer A. Then, 4 µL of 5X nuclease buffer containing or not, nucleases were added and further incubated for 15 min at 30 °C for DNase I or for 30 min at 37 °C for RecJ. 5X RecJ nuclease buffer contains100 mM Tris HCl pH 7.9, 100 mM NaCl, and 25 mM MgCl_2_ and 5X DNase nuclease buffer contains 200 mM Tris HCl pH 8, 50 mM MgSO_4_, and 5 mM CaCl_2_. After addition of loading buffer, samples were loaded onto native polyacrylamide gels as described above.

### Pulsed field gel electrophoresis

*D. radiodurans* exponential phase cultures were concentrated to an *A*_650 nm_ = 20 in 10 mM MgSO_4_ before irradiation. Then, non-irradiated (NI) or irradiated (5 kGy) cultures were diluted in TGY2X or 10 mM MgSO_4_ to an *A*_650 nm_ = 0.2 and incubated at 30 °C. At different post-irradiation incubation times, culture aliquots (5 mL) were removed to prepare DNA plugs as previously described (Harris et al. 2004), except that each agarose embedded DNA plug was digested for 5 h at 37 °C with 1 unit (FDU) of FastDigest *NotI* restriction enzyme before being subjected to pulsed field gel electrophoresis.

## Results and discussion

### The expression of DdrD protein is induced after irradiation and is under control of IrrE/DdrO regulatory proteins

Transcriptomic analyses have previously shown that expression of the *ddrD* gene is induced 8–13 fold after an exposure to 3 kGy of γ rays and 6–9 fold after desiccation (Tanaka et al. 2004). The analysis of the *D. radiodurans* genome predicts that the *ddrD* gene [*dr0326*, in the previous annotation (White et al. 1999) and A2G07_11905 in the new annotation (Hua and Hua 2016)] encodes a protein of 198 amino acids (Mw: 21.200) beginning by an ATG initiation codon (Fig. S1a). The multiple sequence alignment of DdrD proteins from *Deinococcus* genera isolates showed that the *D. radiodurans* DdrD protein exhibits a high similarity with its homologs in *Deinococcus* (> 50% of identity), located predominantly in the first 120 N-terminal amino acids (Fig. S1b).

A potential promoter sequence can be discerned with − 35 and − 10 elements (Fig. S1a). RNA-seq analysis of *Deinococcus deserti,* complemented by proteomic studies showed that the *ddrD, ddrA*, and *ddrC* genes are translated from leaderless mRNA and the TSS (Transcription Start Site) corresponds to the first base of the translation initiation codon (de Groot et al. 2014). It is likely that the *D. radiodurans ddrD* gene is also translated from a leaderless mRNA, lacking the Shine–Dalgarno sequence involved in ribosome binding. It was predicted that, in *D. radiodurans*, 46% of proteins could be translated from leaderless RNA (Zheng et al. 2011), and the authors proposed a correlation between radiation tolerance and leaderless translation initiation. High level of genes without translation leaders in *Deinococcus* species may be important in their adaptation to extreme environmental conditions.

**Fig. 1:**
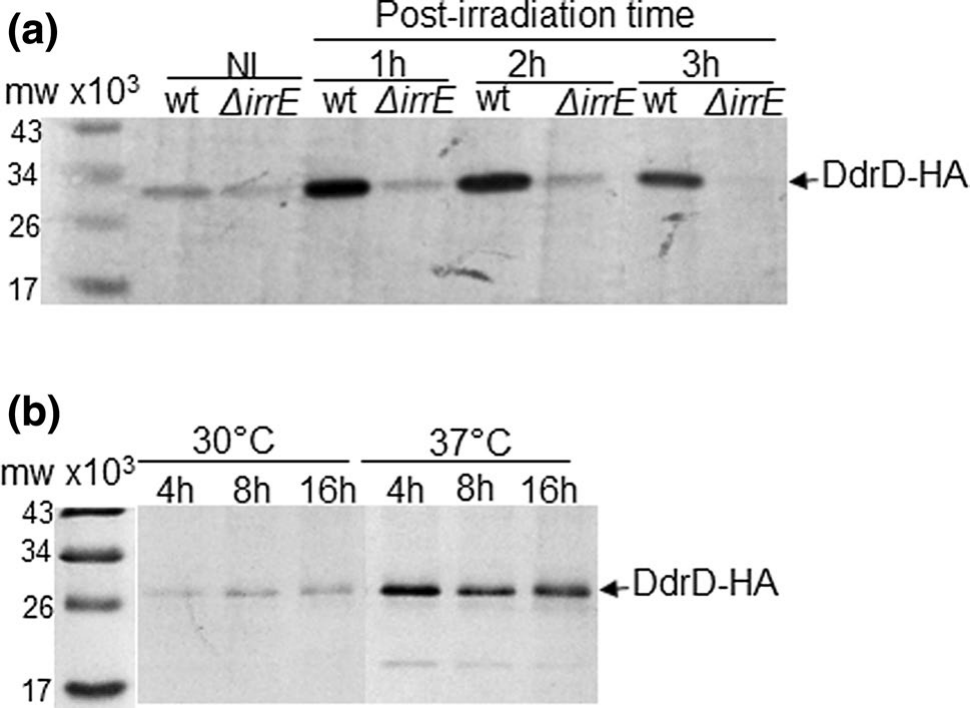
Expression of the DdrD protein is regulated by the IrrE and DdrO proteins. **a** Post-irradiation kinetics of GY15921: *ddrD::HA* (wt) or GY15951: *ddrD::HA* Δ*irrE* (Δ*irrE*) cells exposed to 5 kGy γ-irradiation. After irradiation, the cells were incubated for the indicated times and cell extracts were subjected to SDS–PAGE and analyzed by western blotting with anti-HA antibodies. *NI* non-irradiated cells. **b** GY16922 ([*ddrD::HA-kan ΔddrOΩcat/p11891(prepUTs::ddrO*^+^*)*]) cells grown at 30 °C in TGY2X broth supplemented with spectinomycin to *A*_650_ = 0.3 were harvested and then diluted in a medium without antibiotic and incubated at 30 °C or 37 °C for the indicated times (hours). Cell extracts were subjected to SDS–PAGE and analyzed by western blotting with anti-HA antibodies. Ten µg of protein extract were loaded in each well

An RDRM sequence, the binding site of repressor DdrO (Blanchard et al. 2017; Wang et al. 2015), was found 10 nt upstream of the putative start codon of the *ddrD* gene (Fig. S1a). To test if the expression of the DdrD protein is under the control of the IrrE and DdrO regulator proteins, we analyzed the kinetics of expression of DdrD after exposure to γ-rays in cells lacking IrrE. For this purpose, the DdrD protein was tagged at its C-terminus with the HA epitope and expressed in replacement of the native DdrD protein. Its expression was followed after a 5 kGy γ-irradiation in a wild type strain and in a *ΔirrE* mutant. The presence of the HA-Tag in the C-terminal part of the protein slightly modified its migration on SDS–PAGE (Fig. 1). Western blot analysis showed a basal level expression of DdrD-HA in unirradiated cells that strongly increased after irradiation in wild type cells but it remained constant in the cells devoid of IrrE protein (Fig. 1a). This result shows that IrrE is a positive regulator of *ddrD* gene, correlating with previous transcriptomic approaches (Lu et al. 2012). We also examined the effect of DdrO depletion on the expression of DdrD-HA*.* As *ddrO* is an essential gene (Devigne et al. 2015), we used a *ΔddrO* mutant strain expressing *ddrO* from a prepU_ts_ plasmid and compared the kinetics of expression at 30 °C and at 37 °C, a non-permissive temperature for replication of the plasmid. The depletion of the DdrO protein at 37 °C resulted in an increase of cellular levels of the DdrD-HA protein (Fig. 1b), strongly suggesting that DdrO is a repressor of the expression of DdrD. Taken together, these results confirmed that the DdrD protein belongs to the RDR regulon and that its expression is under the control of the IrrE/DdrO regulatory proteins.

### DdrD binds to ssDNA and to dsDNA with a single-stranded 5′ extension

**Fig. 2:**
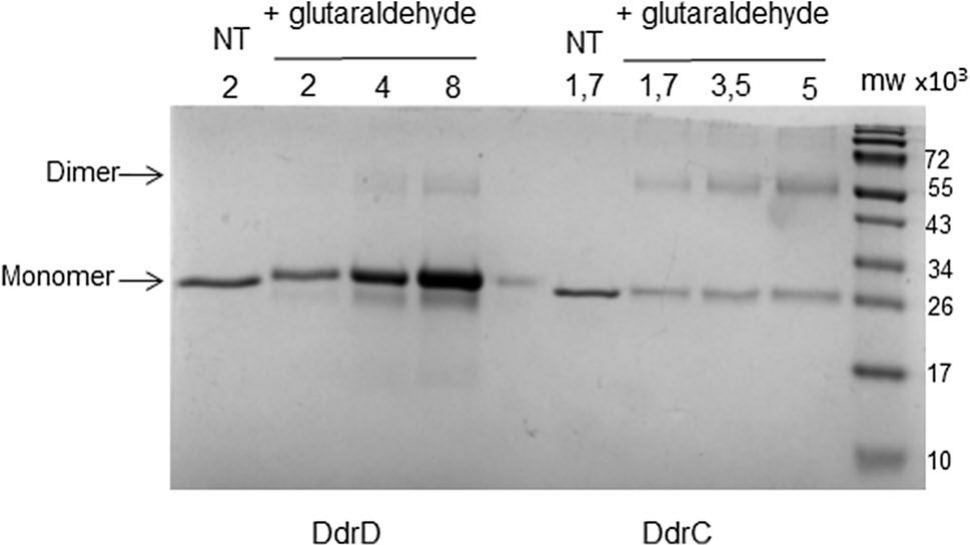
DdrD protein is a monomer in solution*.* Increasing amounts of DdrD or DdrC proteins (µM) were crosslinked with glutaraldehyde. *NT* non-treated protein, *M* molecular weight markers

To analyze the DNA binding properties of DdrD, we first determined the oligomeric state of the native purified DdrD in solution. Previous studies showed that DdrA exhibits a heptameric (Gutsche et al. 2008), DdrB a pentameric (Norais et al. 2009) and DdrC a dimeric structure in solution (de la Tour et al. 2017). As shown in SDS–PAGE analysis (Fig. 2), DdrD migrates approximately to the size deduced from the amino acid sequence. In the presence of glutaraldehyde used as a crosslinking agent, an intense band corresponding to the monomeric form of DdrD was observed on the gel while two bands corresponding both to the monomeric and dimeric forms were observed for DdrC as previously shown (de la Tour et al. 2017). At a DdrD concentration > 4 µM, only faint bands attributed to dimeric forms were visible, indicating that DdrD is mainly present in a monomeric form in solution.

**Fig. 3:**
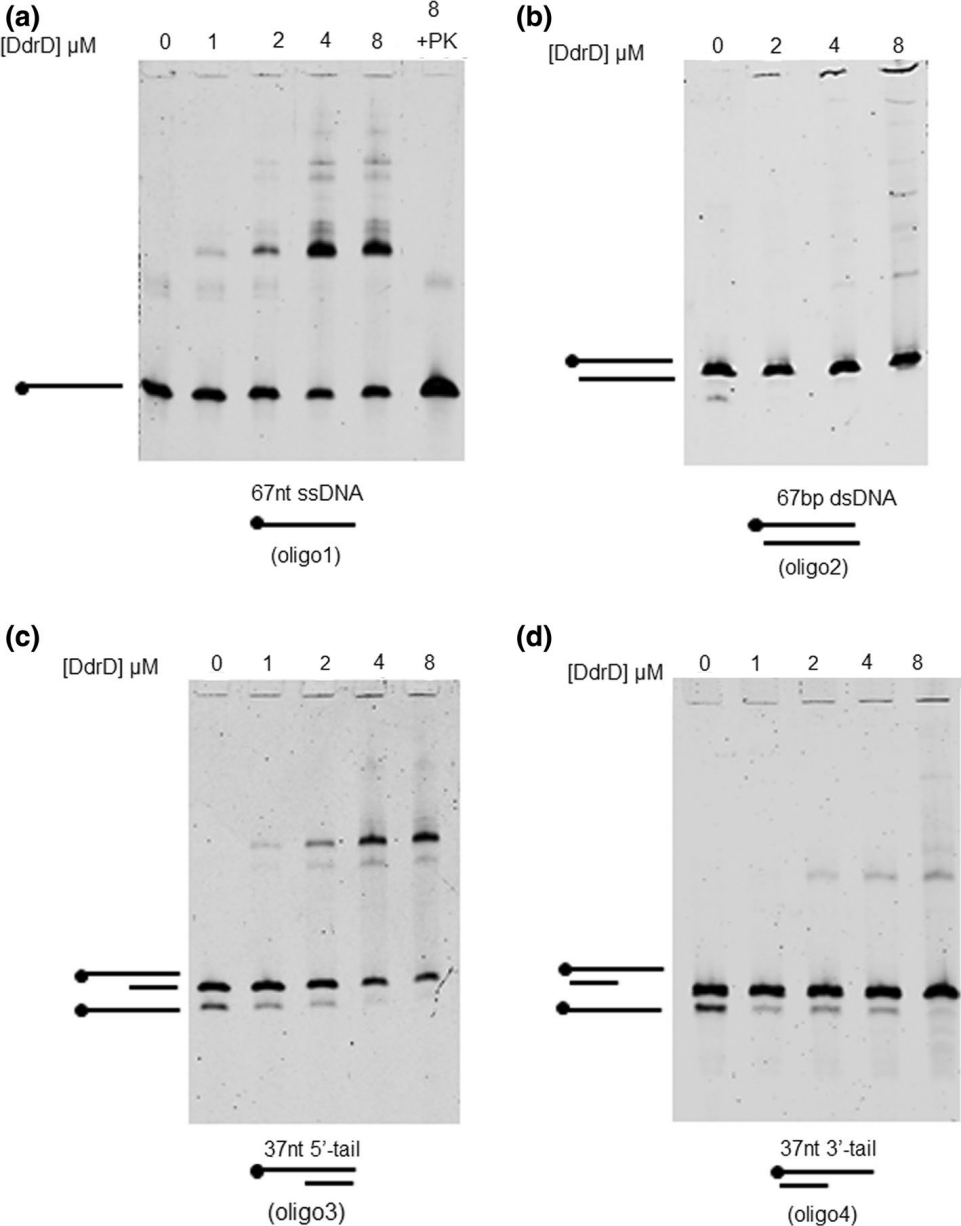
DdrD protein binds to ssDNA and dsDNA with a 5′ extension*.* Increasing concentrations of DdrD were incubated with the indicated DNA substrates (oligos 1, 2, 3, or 4) and the products of the reactions were separated by electrophoresis through 6% native polyacrylamide gels (**a**, **b**, **c**, **d**). On **a**, lane 8 + PK corresponds to the reaction of lane 8 treated with a mixture of 1 mg/mL Proteinase K/0.5% SDS. Lanes 0: DNA controls without DdrD. Dots indicate the position of the Cy5 label

The DNA binding properties of DdrD were then investigated using electrophoretic mobility shifts assays. First, we observed that, contrary to the results obtained with DdrC (de la Tour et al. 2017), no DNA shift was visible when DdrD was incubated with large DNAs such as circular phiX174 ssDNA or linearized phiX174 dsDNA (Fig. S2). However, a DNA shift was observed when a single-stranded 67 mer oligonucleotide (oligo 1) was used as a substrate (Fig. 3a). Faint bands, likely corresponding to larger DdrD/DNA complexes, were observed at 4 and 8 µM but the DNA was not completely shifted even at the highest DdrD concentration. Removal of DdrD by treatment with proteinase K (lane 8 + PK) released the DNA substrate from the nucleoprotein complexes, indicating that the DNA was intact. On the other hand, the corresponding double-stranded 67mer oligonucleotide (oligo 2) was not shifted by DdrD at the same concentrations (Fig. 3b). Thus, DdrD specifically interacts with ssDNA, as previously reported for DdrA (Harris et al. 2008) and DdrB (Norais et al. 2009). DdrC also exhibits a preference for ssDNA (de la Tour et al. 2017).

To further investigate DdrD-ssDNA interactions, we tested DdrD’s ability to bind to a double-stranded oligonucleotide with a single-stranded 5′ or 3′ extension (oligos 3 and 4). When oligo 3 (ds oligonucleotide with a 37-nt 5′ tail) was tested, two shifted bands were visible (Fig. 3c) likely corresponding to both the binding of DdrD to oligo 3 (major band) and to the remaining fraction of non-hybridized ssDNA (minor band) present in the preparation of the substrate and that interacts with DdrD. On the other hand, when oligo 4 (ds oligonucleotide with a 37-nt 3′ tail) was tested, only the faint shifted band corresponding to the DdrD-ssDNA complex was observed (Fig. 3d). Thus, DdrD exhibits some preference for the 5′-ssDNA extension while DdrA preferentially binds to a 3′-ssDNA extension (Harris et al. 2004, 2008) suggesting that the two proteins could protect the DNA ends generated after γ-irradiation.

**Fig. 4:**
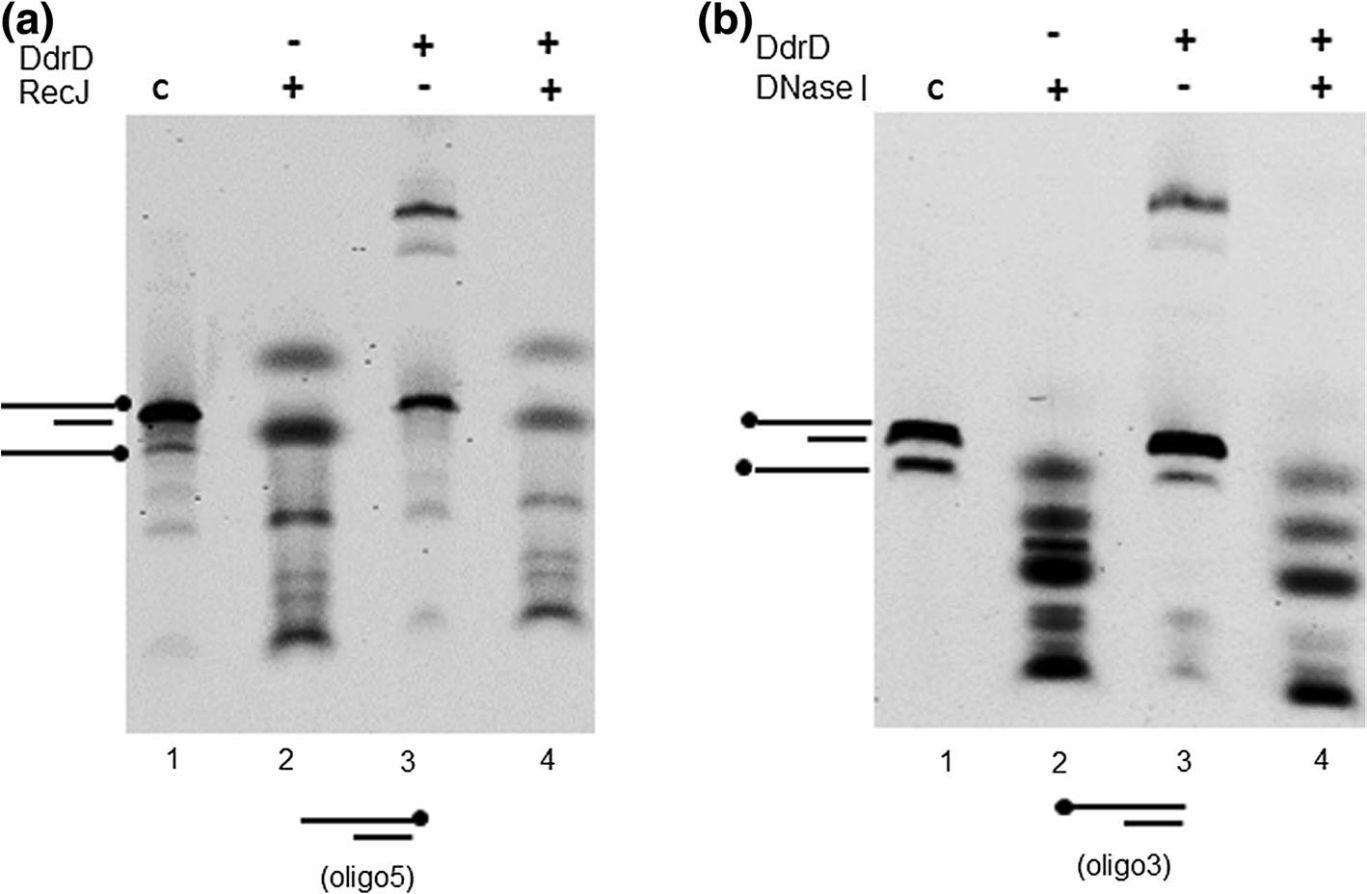
DdrD protein does not protect the 5′ tail of dsDNA from degradation by nucleases*.* The nuclease protection assays were performed using double-stranded oligonucleotide with a 37-nt 5′ tail as the substrate (“Materials and methods”). **a** Ds oligonucleotide labeled at the 3′ end (oligo 5) was incubated with DdrD before addition of RecJ. **b** Ds oligonucleotide labeled at the 5′ end (oligo 3) was incubated with DdrD before addition of DNase I. Lanes 1: DNA controls without protein. Lanes 2: DNA incubation with nuclease alone. Lanes 3: DNA incubation with DdrD alone. Lanes 4: DNA pre-incubated with DdrD 15 min at 4 °C before addition of nuclease

Therefore, we investigated DdrD’s ability to protect the 5′ tail of dsDNA from nucleases (Fig. 4). DdrD protein was incubated either with oligo 5 prior to the addition of RecJ, an exonuclease that digests ssDNA from the 5′ end, or with oligo 3 prior the addition of DNase I, an endonuclease that digests single- and double-stranded DNA. We observed that the presence of DdrD, even at a high concentration (8 µM), does not protect DNA from degradation by RecJ (Fig. 4a, lanes 2 and 4), and only a partial protection could be observed with DNase I (Fig. 4b, lanes 2 and 4). These results suggested that the DdrD does not prevent access of nucleases to DNA. Thus, our in vitro studies showed that DdrD protein binds to ssDNA with a preference for 5′ ends but does not appear to protect DNA from nuclease attack.

### The DdrD protein contributes in vivo to genome recovery after γ-irradiation in nutrient-poor conditions

**Fig. 5:**
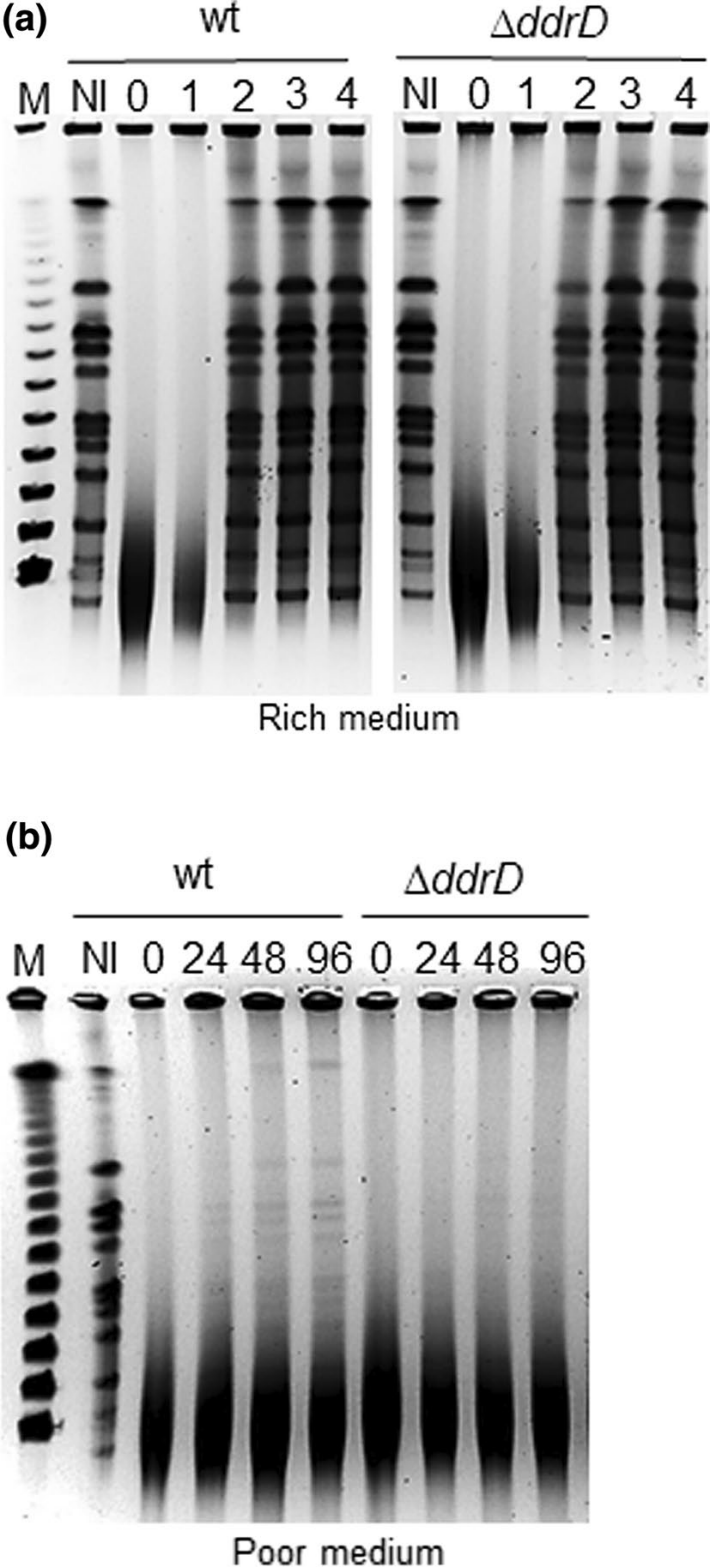
Kinetics of genome reconstitution in wild type and a *ΔddrD* mutant after γ-irradiation. Cells were exposed to 5 kGy γ-irradiation and genome reconstitution was followed by pulsed field electrophoresis of *Not*I treated DNA at the indicated incubation times (hours). **a** After irradiation, cells were incubated in a rich medium (TGY2X). **b** After irradiation**,** cells were incubated in 10 mM MgSO_4_. On each panel, lanes NI control of non-irradiated cells,  M λ DNA fragment ladder (Kb)

To know whether DdrD, like DdrA, plays an in vivo role in the reconstitution of the *D. radiodurans* genome after irradiation, we measured the kinetics of reconstitution of genomic DNA in *ΔddrD* cells exposed to 5 kGy γ-rays. Recovery from damage was monitored by appearance of the pattern of 11 *Not*I digested fragments following irradiation, analyzed by pulsed field gel electrophoresis. When post-irradiation recovery of cultures was followed in a rich medium, the pattern of reconstitution of a *ΔddrD* mutant was identical to that of wild type strain (Fig. 5a). Under these conditions, the genome was reconstituted in approximately 2 h post-irradiation. However, if the cultures were resuspended in 10 mM MgSO_4_ after irradiation, only a partial reconstitution was visible 24 h post-irradiation in the wild type cultures (Fig. 5b), whereas there was no evidence of DNA fragment reassembly in *ΔddrD* cultures at 24 h. From 48 h post-irradiation, a high molecular weight band appeared in wild type cells that was absent in *ΔddrD* cells, indicating that the reconstitution of genomic DNA in cells devoid of DdrD was affected. After 96 h, the DNA reconstruction pattern did not changed in *ΔddrD* cells (Fig. 5b) and remained the same when the post-irradiation time was extended up to 120 h (Fig S3). These observations suggested that DdrD, as observed for DdrA (Harris et al. 2004), contributes to genome reconstitution after irradiation when cultures are incubated under starvation conditions, In a medium devoided of carbon source, the DNA repair mechanisms are much less efficient than in a rich medium, as it is important to protect DNA during genome reconstitution. Although, in vitro, DdrD did not appear to protect DNA from nuclease attack (Fig. 4), it could nevertheless, via its properties of binding to the ssDNA and particularly to 5′ ssDNA extension (Fig. 3) be a part of the *D. radiodurans* genome-protection system composed of the other single-stranded DNA binding proteins, DdrA, DdrB, and DdrC (de la Tour et al. 2017; Harris et al. 2004; Norais et al. 2009; Xu et al. 2010).

### Comparison of the resistance to γ- and UV irradiation of all possible combinations of *ddrA, ddrB, ddrC*, and *ddrD* mutants

**Fig. 6:**
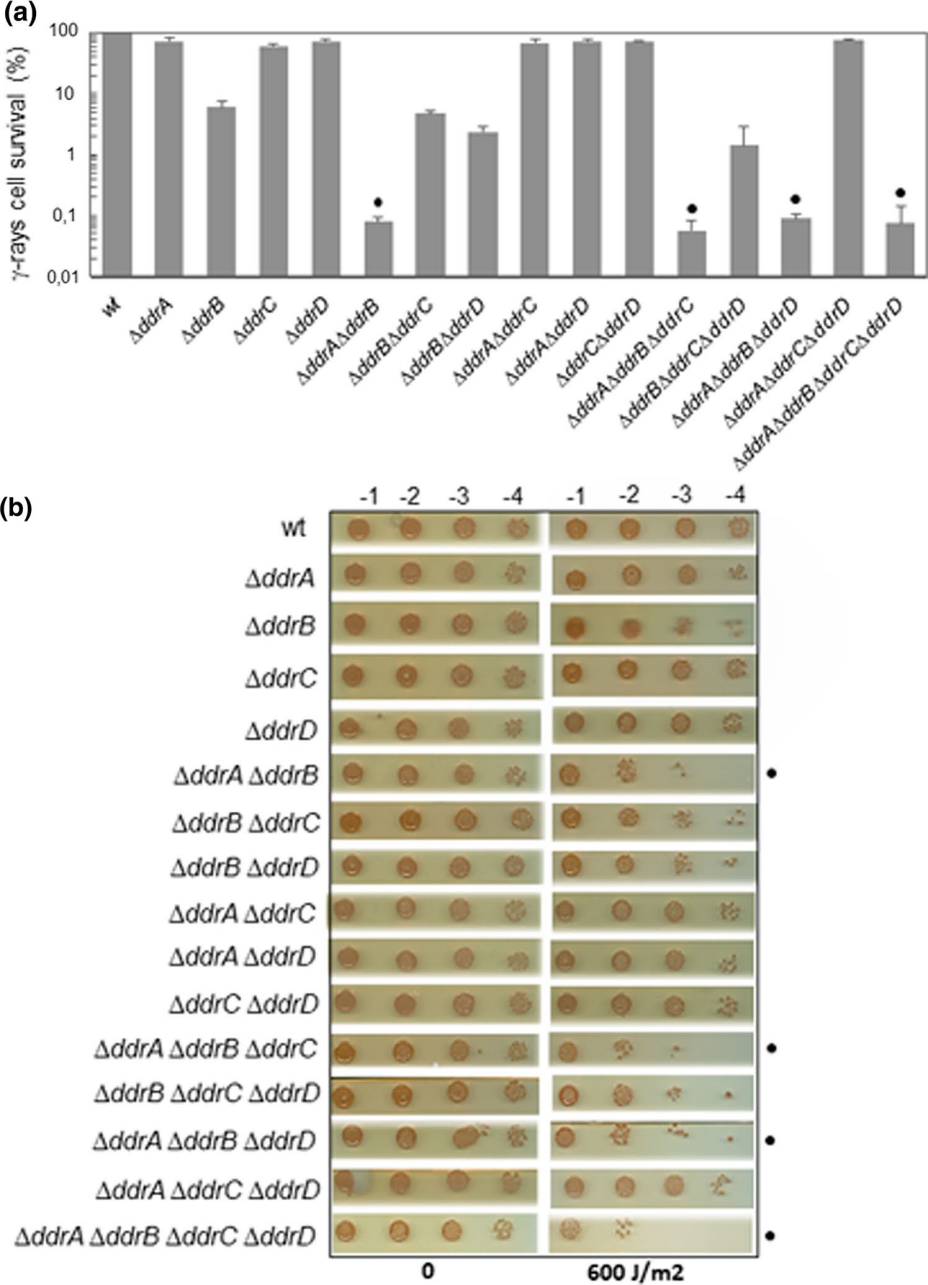
Survival of wild type and mutants generated by deletions of all possible combinations of the *ddrA, ddrB, ddrC,* and *ddrD* genes*.* **a** Survival after exposure to 8 kGy γ-irradiation. The values are the mean of three experiments. They were normalized relative to that of the wild type strain (wt) taken as 100%. **b** Survival after exposure to 600 J m^−2^ UV irradiation. Serial dilutions of cultures were spotted on TGY agar plates and exposed to UV irradiation. On each panel, the dots indicate strains containing deletions of both the *ddrA* and *ddrB* genes

We focused our work on *ddr* genes coding for DNA binding proteins that bind preferentially to ssDNA. Previous studies showed that the *ddrA, ddrB, ddrC*, and *ddrD* genes were among the genes whose expression was most strongly induced in response to stress (Tanaka et al. 2004) but only the deletion of *ddrB* and, at a much lower level that of *ddrA,* reduced the sensitivity of the mutants to γ-irradiation. The absence of one of these genes did not significantly affect the sensitivity to UV radiation (Selvam et al. 2013). Here, we analyzed the response to gamma and UV irradiation of mutant strains generated by deletion of all possible combinations of *ddrA*, *ddrB*, *ddrC*, and *ddrD* genes. We compared cell survival of single, double, triple mutants, and the quadruple mutant (Fig. 6)*.* As previously shown (Tanaka et al. 2004), the single *∆ddrA*, *∆ddrC,* and *∆ddrD* mutants did not exhibit a significant decrease of radioresistance when exposed to 8 kGy gamma irradiation (Fig. 6a). At this dose of irradiation, only the *∆ddrB* mutant was about 15-fold more sensitive than the wild type strain. The survival of double mutants showed that only the double deletion of the *ddrA* and *ddrB* genes resulted in a drastic decrease (about 70-fold) in gamma irradiation resistance of the mutant strain compared to the single *∆ddrB* mutant. The *∆ddrB ∆ddrC* and *∆ddrB ∆ddrD* double mutants exhibited the same sensitivity to this gamma irradiation dose as the *∆ddrB* single mutant*.* The *∆ddrA ∆ddrC* and *∆ddrA ∆ddrD* double mutants had the same sensitivity as the *∆ddrA* single mutant and the *∆ddrC ∆ddrD* double mutant was as resistant as the wild type strain. The addition of *ddrC* or *ddrD* gene deletions in the *∆ddrA ∆ddrB* double mutant did not increase sensitivity to irradiation when compared to the double mutant, and the deletion of *ddrA* or *ddrB* in the *∆ddrC ∆ddrD* double mutant led to a γ-ray sensitivity comparable to that of *∆ddrA* or *∆ddrB* single mutants, respectively. Lastly, the quadruple mutant displayed the same cell survival as the *∆ddrA ∆ddrB* double mutant.

When the different mutant strains were exposed to a dose of 600 J m^−2^ of UV irradiation (Fig. 6b), the *∆ddrA ∆ddrB* double mutant was approximatively tenfold more sensitive to UV than the *∆ddr*B mutant, but the deletion of *ddrC* or *ddrD* genes in the *∆ddrA ∆ddrB* double mutant had no significant effect on the UV sensitivity. A similar response to UV exposure was observed in the quadruple mutant.

These results confirm that the DdrA and DdrB proteins contribute to *D. radiodurans* radioresistance with DdrB playing a major role in this radioresistance. On the other hand, the functions of the DdrC and DdrD proteins remain unclear. Previous studies (de la Tour et al. 2017) showed that a *∆ddrC* mutant was UV sensitive when cells were exposed to UV doses at 750 J m^−2^ and the absence of *ddrC* in *∆uvrA* and *∆uvsE* mutants increased the UV sensitivity of the resulting double mutants. As UvrA and UvsE proteins belong to the UvrABC dependent nucleotide excision repair and UVDE repair pathways (Moseley and Evans 1983), respectively, it was suggested that DdrC could be involved in DNA repair of highly UV-damaged DNA. However, such a role could not be attributed to DdrD because the *∆ddrD* mutant exhibited a UV sensitivity comparable to that of the wild type strain at 750 J m^−2^ and the *ddrD* deletion in *∆uvrA* and *∆uvsE* mutants had no effect on the UV sensitivity (Fig. S4). Finally, we tested if the *ddrD* deletion increased UV sensitivity of a *pprA* mutant, as previously described (Selvam et al. 2013), but in our hands, the UV sensitivity of the *∆pprA ∆ddrD* double mutant was comparable to that of the *∆pprA* single mutant (Fig. S4), indicating that the activities of PprA and DdrD do not overlap in vivo.

### The absence of the *ddrD* gene in the *∆ddrB* mutant partially restores the frequency of plasmid transformation

**Fig. 7:**
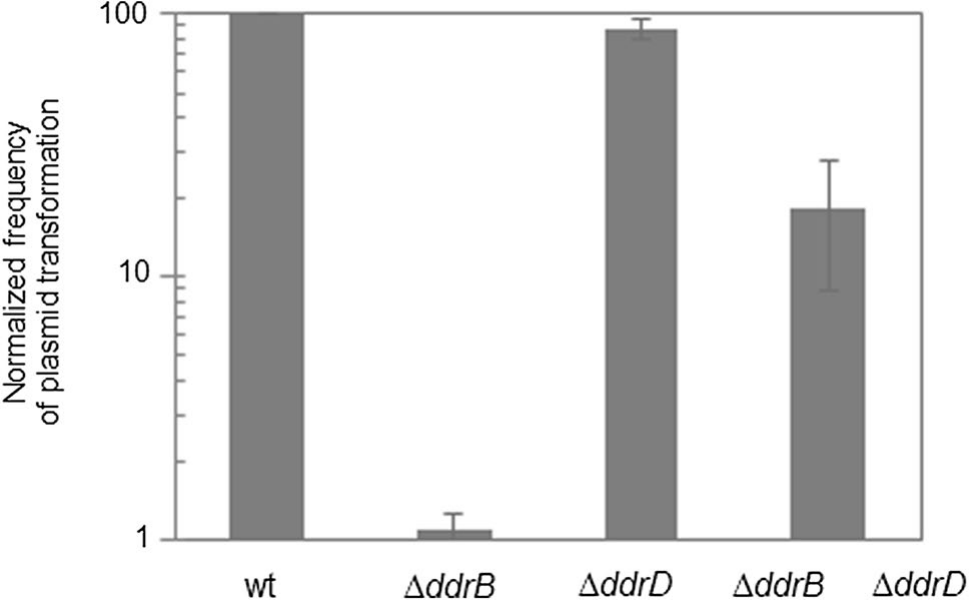
Frequencies of plasmid transformation in single *∆ddrB, ∆ddrD,* and *∆ddrB ∆ddrD* double mutants*.* Bacteria were transformed with 200 ng p11559 plasmid (conferring spectinomycin resistance). Transformation frequencies were expressed as the number of spec^R^ transformants divided by the total number of viable cells in the transformation mixture. The values obtained were normalized relative to that of the wild type strain, taken as 100. The results are the mean of at least three experiments

The *D. radiodurans* bacterium, characterized by its extreme radioresistance, is also naturally competent. In this process, DNA is translocated as ssDNA into the cytosol and protected from degradation by ssDNA binding proteins prior integration into the chromosome by homologous recombination or reconstituted to form an autonomous plasmid (Kruger and Stingl 2011). In bacteria, several ssDNA binding proteins (SSB) as SSB, DprA, RecA, and RecO protect internalized ssDNA from degradation by nucleases (Kidane et al. 2012). The establishment of plasmid DNA requires a single strand annealing to pair internalized complementary plasmid DNA fragments to reconstitute a circular replicon in naturally transformable bacteria such as *Streptococcus pneumoniae* and *Bacillus subtilis* (Kidane et al. 2009; Saunders and Guild 1981). In *B. subtilis*, RecO and DprA mediate annealing of two complementary strands (Yadav et al. 2013) while in *D. radiodurans*, RecO seems to play a minor role in plasmid transformation when DdrB is present in the cells (Ithurbide et al. 2020). DdrB, through its ability to bind to ssDNA and SSB-like properties, participates in the protection of internalized ssDNA (Ithurbide et al. 2020). It was also previously showed that cells devoid of DdrB were affected in the establishment of plasmid DNA during natural transformation in *D. radiodurans* (de la Tour et al. 2011) suggesting that DdrB likely participates to the plasmid reconstruction through its single strand annealing activity. Here, we tested whether the absence of the *ddrD* gene would affect the frequency of plasmid transformation in *D. radiodurans.* We observed that the frequency of transformation of plasmid DNA in the *∆ddrD* mutant was the same as in the wild type strain (Fig. 7). However, while the frequency of transformation by plasmid DNA decreased approximately 90-fold in the single *∆ddrB* mutant compared to the wild type strain, it decreased only approximately 18-fold in the *∆ddrB ∆ddrD* double mutant. Thus, the absence of DdrD partially restored the frequency of transformation of the *∆ddrB* mutant indicating that some proteins were able to reconstruct the plasmid in the absence of DdrB and DdrD. We suggest that DdrD, through its ability to bind ssDNA, could partially prevent the actors of plasmid transformation such as RecO or DprA from reconstructing an intact plasmid from ssDNA fragments in the absence of DdrB. The transformation process in a *∆ddrB ∆ddrD* double mutant would, therefore, be more efficient than in a *∆ddrB* single mutant.

## Conclusion

In this study, we showed that DdrD belongs to the family of ssDNA binding proteins including the deinococcal specific Ddr proteins, DdrA, DdrB, DdrC, whose expression is highly induced following γ-irradiation. The *ddrD* gene expression is controlled by the IrrE/DdrO protein pair, a very efficient regulation system known in *D. radiodurans*. Although the redundant activities of these Ddr proteins make it difficult to assign a precise role to each of them*,* we propose that DdrD protein, through its ability to bind to ssDNA as well as to 5′ overhang DNA ends, helps cells to recover from DNA damage when *D. radiodurans* is exposed to an extensive genotoxic stress*.* Moreover, like other ssDNA binding proteins, DdrD might also regulate efficiency of transformation in this bacterium.

## Supplementary Information

Below is the link to the electronic supplementary material.

Supplementary file1 (PDF 1454 KB)
