# Supplementary material for: Characterization of the DdrD protein from the extremely radioresistant bacterium Deinococcus radiodurans
Source: Extremophiles. 2021 May 29;25(4):343–55. doi: 10.1007/s00792-021-01233-0 (PMC8254717; doi:10.1007/s00792-021-01233-0)
Supplement: Supplementary file 1 — Supplementary file1 (PDF 1454 KB) [file 792_2021_1233_MOESM1_ESM.pdf]

Supplementary material

Article title: Characterization of the DdrD protein from the extremely radioresitant bacterium *Deinococcus radiodurans*

Journal name: Extremophiles

Author names: Claire Bouthier de la Tour, Martine Mathieu, Pascale Servant, Geneviève Coste, Fabrice Confalonieri

Affiliation: Université Paris-Saclay, CEA, CNRS, Institute for Integrative Biology of the Cell (I2BC), 91198, Gif sur Yvette, France

Corresponding author e-mail adress: claire.bouthier@i2bc.paris-saclay.fr

a

```
  1  gtgcagtaag tgcagggcta aaaaactcag gagatgaggg gcgcaggcca

 51  gttggcggct gcgcccctct cctgactcgc cttcacattt caagaatctt

101  cttgacaatt ctgctaaaaa cagaataata gagctatgga taccctgaaa
       -35          RDRM        -10
                                         >>....DdrD.....>
                                           m   d  t  l  k

151  aaagctggaa cgatgctcgc ccacctcgac ctctttcact cgatgctcga
     >.......................DdrD.......................>
       k  a  g   t  m  l   a  h  l  d   l  f  h   s  m  l
```

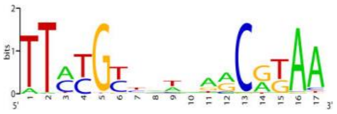

RDRM consensus

b

```
radiodurans      MDTL KKAGTMLAHLDLFHSMLDLRRLLQLAAYMKERGDRAMLISAGEITLIGSESMTAP
wulumuqiensis    MDTL KKAGAMLPHLDLFQSMLDLRRLLQLAAHMKERGDRAMLISGTEITLIGGESLSAP
proteolyticus    MDNLLKKAGAMLPHLELFGHMASLRGLLQLAAHMEERGDRVTLISPEAITLVGSDMTTDP
marmoris         MDTL KKAGAMLAHLDLFHQMLDLRGLLQLAAHMEERGDRVTLISGETITLIGAEMLSDA
gobiensis        MDTL KKAGAMLQHLDLFHTMLDLRGLLQLAAHMEERGDRVTLISPEQITLIGGAMQADA
deserti          MDTL KKAGAMLAHLDLFTRMRDLRSLLQLAAHMEERGDRVTLISPDNITLVGTEMSAQP
geothermalis     MDTLLKKAGAMLAHLDLFTHMLHLRGLLQLAAHMEERGDRVTLISPELITLVGAEMTSDA
Consensus        MDtL.KKAGAMLaHL#LF..MldLRgLLQLAAHMeERGDRvtLISpe.ITL!G.em..dp
                 ** * **** ** ** **  *  ** ****** * *****  ***   *** *

radiodurans      EVVTSKGETIDAATAYRVLGQLEGYEAPEYAVNREALAALNARAVAELEGSEALRAFGDT
wulumuqiensis    EIVTSKGETVDAATAYRVLGQLEGYDAPEYAVNREALAALNARAVAELEGSEALRAFGET
proteolyticus    VIHTSKGATVTAEGAYTLMHTLKGHEAPEYAVTREELKALNARAVADIEAGPALAAFGET
marmoris         GVTTGKGARIEAATAYRVLQGLKGHDAPEYAVTREELGALNARAVAELEGGDALKAFGDT
gobiensis        RVTTSKGATIESGTAYRVLHRLKGHEAPEYAVTREELGALNARAVADLESGDALRAFAET
deserti          ALTTAKGATIEARTAYSVLQGLKGHEAPEYAVTREELGALNARAVADLESSDALRAFADT
geothermalis     SVTTSKGATIEAGTAYRVLRTLKGHDAPEYAVTREELKALNARAVAELEASEAMRAFGET
Consensus        .vtTsKGat!ea.tAYrv$..LkGh#APEYAVtREeL.ALNARAVA#lE.s.A$rAFg#T
                    * **       **     * *  ****** ** * ********  *   *  **  *

radiodurans      LARISAAPTDPAGPERPGTDRAERTAAERTASERATHDRASTERPARPRRSAEPEAVRTE
wulumuqiensis    LARISAAPTDPAGPERPGTDRA        ASERAAAERATSERSARPRRTPDAETPRSE
proteolyticus    LARLGLGDAGSAGSARPEVQVQ    AETQAEAPAPVTPAEEPRPERTSRSRRAEA QAE
marmoris         LARIGAAPAAAPAP                AQAPQPEASAPAERPGRGRRAAETEENTT
gobiensis        LTRVSAAPATAPA                        GDAAAERPARARRAPEGEAQPAT
deserti          LTRIGAAPAATPT                         DAPTERPARARRGEVSEPAVS
geothermalis     LARIGVPAAGSGAP                       VEAVAERPARNRRAAEAEGA
Consensus        LaRigaapa....p........    ....a........A..eRPaR.rR..e.E.....
                 * *                                    *   *  *  *    *

radiodurans      DAPQPNAEASEAGENTPAA
wulumuqiensis    AAEQPTDDSAPAA
proteolyticus    AAEVPAAS
marmoris           EQPAA
gobiensis        PGEQPAA
deserti            EQPAA
geothermalis      GEQPAA
Consensus        ..EqPaA......
                     *
```

Fig. S1: Promoter region of the *D. radiodurans ddrD* gene (a) and multiple sequence alignment of DdrD proteins from *Deinococcus* (b). **a**. Potential -35 and -10 elements, predicted from the BPROM program Softberry, are underlined and the RDRM motif is written in red. The RDRM consensus sequence is shown on the right of the panel (from Makarova, 2007)[4]. **b**. Identical residues are in red and stars represent the consensus sequence.

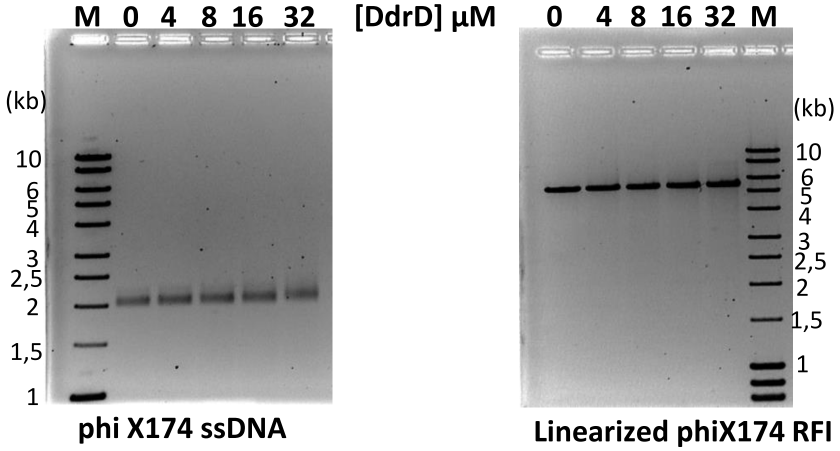

Fig. S2: DdrD protein does not bind to large DNAs. Protein - DNA binding was analyzed by EMSA on a 1,2% agarose gel. 200 ng of each DNA (corresponding to 3 nM phiX174 ssDNA and 6 nM linearized phiX174 RFI) were incubated in 20 µl of a buffer containing 40 mM Tris-HCl pH 7.8, 5 mM MgCl2, 1.5 mM DTT, 50 mM NaCl, 12% glycerol, with increasing concentrations of DdrD as indicated in the figure. Electrophoreses were performed in TEP buffer (36 mM Tris-HCl, pH 7.8, 30 mM NaH_2PO_4, 1 mM EDTA) at 4.3V/cm for 3 h at 4°C. After staining of the gels with ethidium bromide (1 µg/ml), bands were visualized under UV, using Image Lab (Bio-Rad) software.

M: Molecular weight markers.

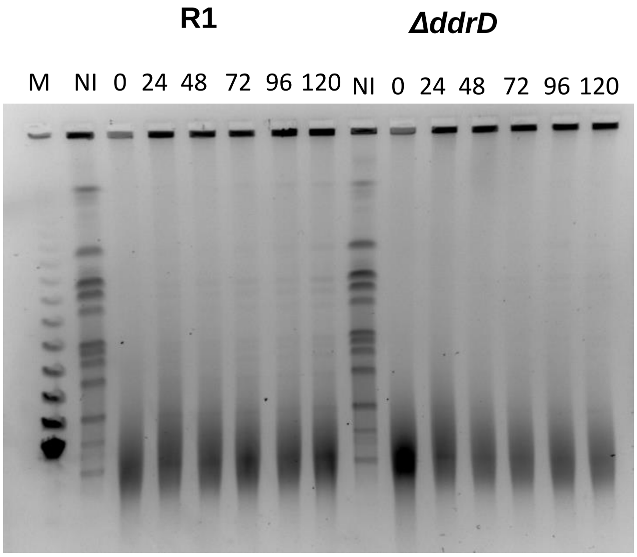

Fig. S3. Pattern of genome reconstitution in wild type and *ΔddrD* mutant after 120h post irradiation time in $MgSO_4$. Cells were exposed to 5 kGy γ-irradiation and genome reconstitution was followed by pulsed field electrophoresis of *Not*I treated DNA at the indicated incubation times (hours). After irradiation, cells were incubated in 10 mM $MgSO_4$. lanes NI: control of non-irradiated cells. M: λ DNA fragment ladder (Kb)

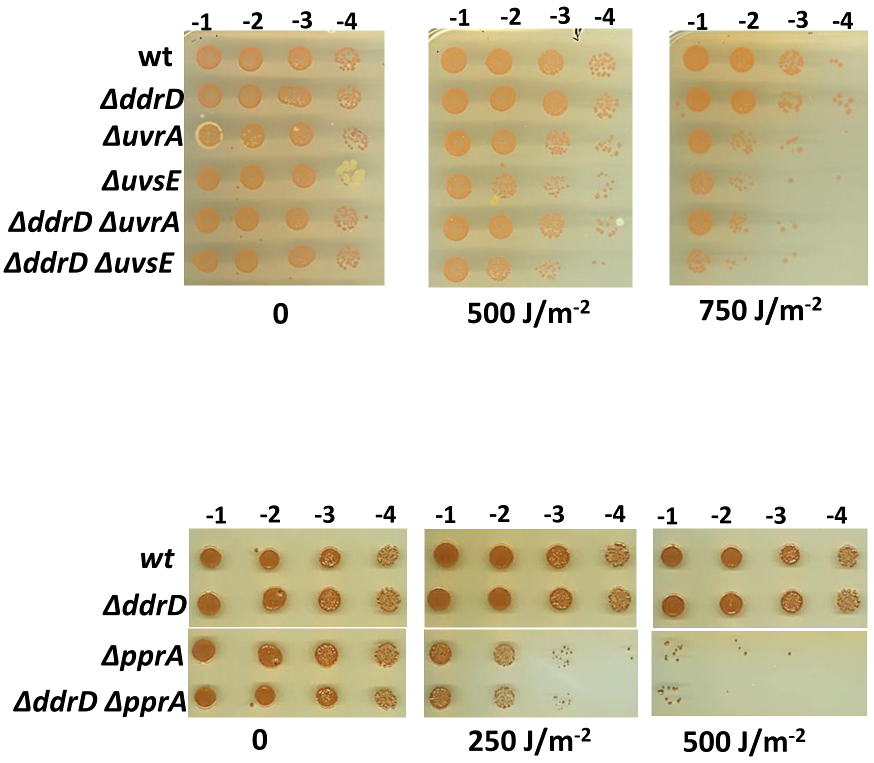

Fig. S4: **The absence of DdrD does not increase UV sensitivity of *ΔuvrA* , *ΔuvsE* and *ΔpprA* mutants**.

Cultures of wild type (wt), *ΔddrD*, *ΔuvrA*, *ΔuvsE*, *ΔddrD ΔuvrA*, *ΔddrD ΔuvsE*, *ΔpprA*, *ΔddrDΔpprA* were serially diluted in TGY2x broth and aliquots (10 µl) of each dilution were spotted on TGY agar plates. Then, the plates were exposed to indicated doses of UV irradiation before incubation at 30°C for 3-5 days.
